# Genetic support of the causal association between gut microbiome and COVID-19: a bidirectional Mendelian randomization study

**DOI:** 10.3389/fimmu.2023.1217615

**Published:** 2023-07-07

**Authors:** Zengbin Li, Guixian Zhu, Xiangye Lei, Liqiong Tang, Guangyao Kong, Mingwang Shen, Lei Zhang, Lingqin Song

**Affiliations:** ^1^ Department of Oncology, The Second Affiliated Hospital of Xi’an Jiaotong University, Xi’an, China; ^2^ China-Australia Joint Research Center for Infectious Diseases, School of Public Health, Xi’an Jiaotong University Health Science Center, Xi’an, China; ^3^ National and Local Joint Engineering Research Center of Biodiagnosis and Biotherapy, The Second Affiliated Hospital of Xi’an Jiaotong University, Xi’an, China; ^4^ Melbourne Sexual Health Centre, Alfred Health, Melbourne, VIC, Australia; ^5^ Central Clinical School, Faculty of Medicine, Nursing and Health Sciences, Monash University, Melbourne, VIC, Australia

**Keywords:** gut microbiota, SARS-CoV-2, COVID-19, Mendelian randomization, causality, rct, randomized controlled trial

## Abstract

**Background:**

The association between gut microbiome and coronavirus disease 2019 (COVID-19) has attracted much attention, but its causality remains unclear and requires more direct evidence.

**Methods:**

In this study, we conducted the bidirectional Mendelian randomization (MR) analysis to assess the causal association between gut microbiome and COVID-19 based on the summary statistics data of genome-wide association studies (GWASs). Over 1.8 million individuals with three COVID-19 phenotypes (severity, hospitalization and infection) were included. And 196 bacterial taxa from phylum to genus were analyzed. The inverse-variance weighted (IVW) analysis was chosen as the primary method. Besides, false discovery rate (FDR) correction of *p*-value was used. To test the robustness of the causal relationships with *p*-FDR < 0.05, sensitivity analyses including the secondary MR analyses, horizontal pleiotropy test, outliers test, and “leave-one-out” analysis were conducted.

**Results:**

In the forward MR, we found that 3, 8, and 10 bacterial taxa had suggestive effects on COVID-19 severity, hospitalization and infection, respectively. The genus *Alloprevotella* [odds ratio (OR) = 1.67; 95% confidence interval (95% CI), 1.32–2.11; *p* = 1.69×10^−5^, *p*-FDR = 2.01×10^−3^] was causally associated with a higher COVID-19 severity risk. In the reverse MR, COVID-19 severity, hospitalization and infection had suggestive effects on the abundance of 4, 8 and 10 bacterial taxa, respectively. COVID-19 hospitalization causally increased the abundance of the phylum *Bacteroidetes* (OR = 1.13; 95% CI, 1.04–1.22; *p* = 3.02×10^−3^; *p*-FDR = 2.72×10^−2^). However, secondary MR analyses indicated that the result of COVID-19 hospitalization on the phylum *Bacteroidetes* required careful consideration.

**Conclusion:**

Our study revealed the causal association between gut microbiome and COVID-19 and highlighted the role of “gut-lung axis” in the progression of COVID-19.

## 1 Introduction

The COVID-19 pandemic, caused by the severe acute respiratory syndrome coronavirus 2 (SARS-CoV-2), is imposing significant economic and healthcare challenges on society and is expected to do so for the foreseeable future (1, 2). While SARS-CoV-2 is known to mainly infect respiratory tract, increasing evidence suggests its potential involvement in the pathogenesis of COVID-19 via the gastrointestinal tract (3, 4). In addition to its ability to infect and replicate in intestinal enterocytes (5), SARS-CoV-2 can induce the upregulation of angiotensin converting enzyme-2 receptor expression in intestinal epithelial cells (6, 7). Frequent occurrence of gastrointestinal symptoms has been observed in individuals with SARS-CoV-2 infection (8), and a meta-analysis indicated that those with gastrointestinal involvement have an increased risk of severe disease (9).

Emerging evidence has shed light on the connection between the gut microbiome and the pathogenesis of COVID-19, through a mechanism known as the “gut-lung axis” (3, 10, 11). The gastrointestinal tract is considered to be the largest organ of the human immune system (12). Epidemiological studies have indicated that SARS-CoV-2 infection could result in alterations of gut microbiome (13–16), and the prognosis of COVID-19 appeared to be closely linked to the composition of gut microbiome (17–19). Previous studies have suggested that the resident microbiota in the gastrointestinal tract played a critical role in regulating host immunity, thereby providing defense against SARS-CoV-2 infection (20–22). Probiotics, a beneficial group of microorganisms, are known for their effectiveness in restoring homeostasis of gut microbiota, enhancing immunity, and exhibiting antiviral potential (3, 23). Clinical trials have demonstrated that probiotic supplements could restore the homeostasis of gut microbiota, potentially leading to improved prognosis of COVID-19 (24–26). These evidences suggested that the gut microbiome might be a target for the prevention, diagnosis and treatment of COVID-19. However, the association between gut microbiome and COVID-19 was not well-established because it could be easily influenced by unmeasured confounders (27–29). Furthermore, the association was susceptible to unavoidable biases and reverse causation (30).

Randomized controlled trials are considered the benchmark for investigating the causal link between gut microbiome and COVID-19. Regrettably, the screening of gut microbiome for early diagnosis and prognosis of COVID-19 is currently limited owing to the impact of external factors, such as research methods and technology (30). Additionally, a substantial amount of human and material resources are required to conduct the randomized controlled trials, resulting in a burdensome workload. In these circumstances, MR analysis has been advocated as an emerging approach (31). MR analysis is an alternative approach to assess the causal link between exposure and outcome. This method utilizes genetic variants as instrumental variables (IVs) that are randomly distributed during meiosis as unconfounded surrogates for the exposure (32). MR is analogous to the random assignment of interventions in the randomized controlled trials and can thus address the issues of reverse causation and confounders that are commonly found in nonrandomized studies (30). Owing to these strengths, MR analysis has been extensively used to identify factors associated with COVID-19 (28, 33–36).

In the study, utilizing the bi-directional MR approach, we evaluated both the causal impacts of gut microbiome on COVID-19 phenotypes and the causal impacts of COVID-19 phenotypes on gut microbiome. Our objective was to clarify the involvement of gut microbiome in the diagnosis and prognosis of COVID-19, with the ultimate goal of promoting the development of novel strategies, including probiotic therapy, fecal microbiome transplantation, and antimicrobial stewardship.

## 2 Methods

### 2.1 Study design

**Figure 1:**
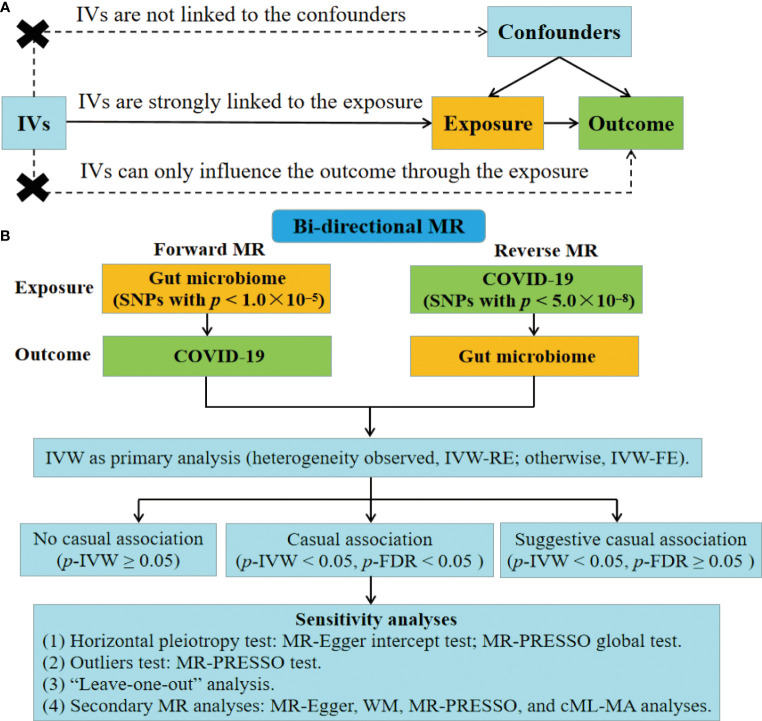
Fow diagram of the study. **(A)** Schematic of the MR design. **(B)** Overview of the bi-directional MR study. IVs, instrumental variables; FE, fixed-effect; RE, random-effect; FDR, false discovery rate; WM, weighted median; MR-PRESSO, MR pleiotropy residual sum and outlier; cML-MA, constrained maximum likelihood and model averaging-based.

The MR analysis is based on the following assumptions (**Figure 1A**) (30). (1) IVs are strongly linked to the exposure. (2) IVs are not linked to the confounders. (3) IVs can only influence the outcome through the exposure, without involving alternative pathways. **Figure 1B** depicts the study design for examining the causal link between gut microbiome and COVID-19 using the bi-directional MR analysis. We first selected gut microbiome as the exposure and COVID-19 as the outcome to detect whether the gut microbiome prevented or promoted the occurrence of COVID-19. To explore the changes in gut microbiome after the occurrence of COVID-19, we conducted a reverse MR analysis (COVID-19 as the exposure; gut microbiome as the outcome). This MR study followed the Strengthening the Reporting of Observational Studies in Epidemiology-Mendelian Randomization (STROBE-MR) guidelines (**Table S1**) (37).

### 2.2 Data sources

GWAS summary data on the human gut microbiome was obtained from the largest multi-ethnic GWAS meta-analysis conducted by the MiBioGen consortium (38). It included 18,340 participants from twenty-four cohorts. Data of gut microbiome was generated by targeting the 16S ribosomal RNA gene, specifically the V4, V3-V4, or V1-V2 regions, and primarily using the Illumina sequencing platform (39). Microbiota quantitative trait loci mapping analysis was conducted to investigate the interactions between host genetic variants and gut microbiome. In addition, the covariate-adjusted abundance of gut microbiome was analyzed, considering factors such as age, sex, technical variables, and principal components (38). Data of gut microbiome covered 211 taxa whose mean abundance more than 1%, encompassing 131 genera, 35 families, 20 orders, 16 classes, and 9 phyla. 196 bacterial taxa were ultimately included in the MR analysis after excluding 15 taxa belonging to unknown groups (12 genera and 3 families). More detailed information about the GWAS of gut microbiome can be found in the literatures (38, 39).

The GWAS summary data on COVID-19 was obtained from the global COVID-19 Host Genetics Initiative, with the exception of the “23andMe” dataset (40). The researchers had already adjusted the original data for covariates, including age, sex, age^2^, age × sex, and principal components. The MR study included 1,683,768 participants, with 38,984 infected cases and 1,644,784 un-infected individuals, for COVID-19 infection analysis. 1,887,658 participants, with 9,986 hospitalized cases and 1,877,672 un-infected individuals, for COVID-19 hospitalization analysis. 1,388,342 participants, with 5,101 severe cases and 1,383,241 un-infected individuals, for COVID-19 severity analysis. Individuals who died or required respiratory assistance as a result of COVID-19 infection were classified as severe cases (40).

### 2.3 Selection of IVs

Single nucleotide polymorphisms (SNPs) were used as IVs based on these criteria. (1) In the forward MR analysis, since few SNPs of gut microbiome met a *p* less than 5.0×10^–8^, we relaxed the *p*-value threshold (**Figure 1B**). SNPs with *p* lower than 1.0×10^–5^ were selected as IVs, following previous studies (41–47). In the reverse MR analysis, SNPs of COVID-19 with *p <* 5.0×10^–8^ were used (**Figure 1B**). (2) We applied clumping to restrict SNPs with low linkage disequilibrium (r^2^ less than 0.001; genetic distance = 10,000 kb) (32). (3) Palindromic SNPs were removed. (4) Only SNPs with minor allele frequency (MAF) more than 0.01 were included. (5) We also calculated F-statistics for the SNPs to assess their instrumental strengths. $F=\frac{\beta^{2}}{SE^{2}}$ . SNPs with an F-statistic < 10 would be removed (48).

### 2.4 Statistical analysis

We conducted the bi-directional MR analysis to assess the causal impacts of gut microbiome on COVID-19 phenotypes and the causal impacts of COVID-19 phenotypes on gut microbiome (**Figure 1B**). IVW analysis was selected as the primary method complying with the STROBE-MR guidelines (37). It employs a meta-analysis method that combines the Wald ratio to provide the casual estimate. IVW analysis is considered precise and robust because it utilizes information from all IVs (32, 49). In addition, the heterogeneity was evaluated using Cochran’s Q test. If no heterogeneity was observed (Q_*p*-value < 0.05), the fixed-effect (FE) model of IVW was utilized. Alternatively, a random-effect (RE) model of IVW was applied (30, 32). The OR and corresponding 95% CI were reported as the results of the bi-directional MR analysis. *P*-value < 0.05 was considered was considered statistically significant. Besides, we conducted the FDR correction (*p-*FDR) with the threshold of 0.05. A causal association was considered significant when the IVW approach yielded a *p*-FDR < 0.05. In addition, we defined a suggestive association as having a *p <* 0.05 but a *p*-FDR ≥ 0.05 with the IVW approach (**Figure 1B**).

To assess the robustness of the findings related to causal relationships with a significance level of *p-*FDR < 0.05, several sensitivity analyses were conducted (**Figure 1B**). First, we employed the MR-Egger test (50) and MR pleiotropy residual sum and outlier (MR-PRESSO) global test (51) to identify horizontal pleiotropy. Second, the MR-PRESSO test was conducted to test for outliers of the SNPs. Third, we employed the “leave-one-out” analysis to assess the potential influence of individual SNP on the MR effect. Furthermore, we performed secondary MR analyses including the MR-Egger analysis (52), weighted median (WM) (53) analysis, MR-PRESSO analysis (51), and constrained maximum likelihood and model averaging-based (cML-MA) analysis (54). We considered a causal association to be authentic only when all MR methods indicated the same direction of effect. MR-Egger is capable of identifying certain deviations from the standard IV assumptions, and producing an effect estimate that is not affected by such deviations (52). WM can yield a causal estimate even when half of the information is derived from invalid IVs (53). cML-MA is a MR method that uses constrained maximum likelihood and model averaging, and has been shown to be resilient against both correlated and uncorrelated pleiotropy while maintaining a low type-I error rate (54). “TwoSampleMR” (55), “MRPRESSO” (51), and “MRcML” (54) were the primary R packages utilized in the study. All analyses were conducted using the R v4.1.2 (R Foundation, Vienna, Austria).

### 2.5 Ethical approval

This study analyzing publicly available summary-level data was exempt from ethical approval.

## 3 Results

### 3.1 Causal effects of gut microbiome on COVID-19 phenotypes

In the forward MR (gut microbiome as the exposure), we identified 2148, 2127, and 2137 SNPs associated with gut microbiome for COVID-19 severity, hospitalization, and infection, respectively. F-statistics greater than 10 for all SNPs related to gut microbiome indicated the absence of the weak instrument bias (**Table S2**). The adjusted IVW results after accounting for heterogeneity are presented in **Table S3**.

**Figure 2:**
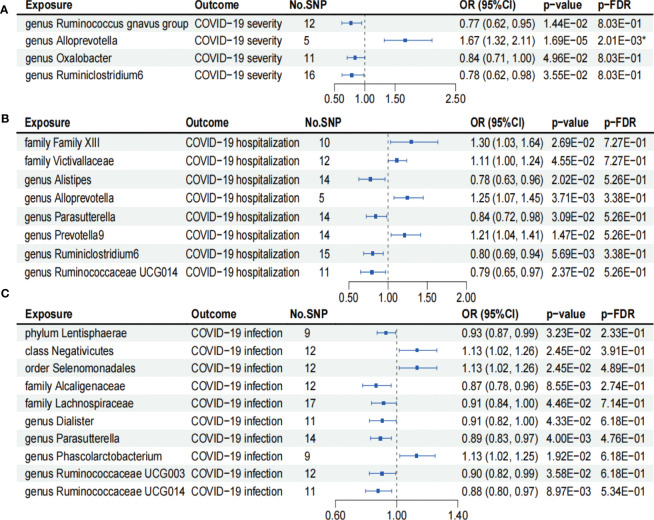
Forests plot of causal effects of gut microbiome on COVID-19 phenotypes (*p*-IVW > 0.05). **(A)** COVID-19 severity. **(B)** COVID-19 hospitalization. **(C)** COVID-19 infection. FDR, false discovery rate. * *p*-FDR < 0.05. E-01 represents 10^−1^.

**Figure 3:**
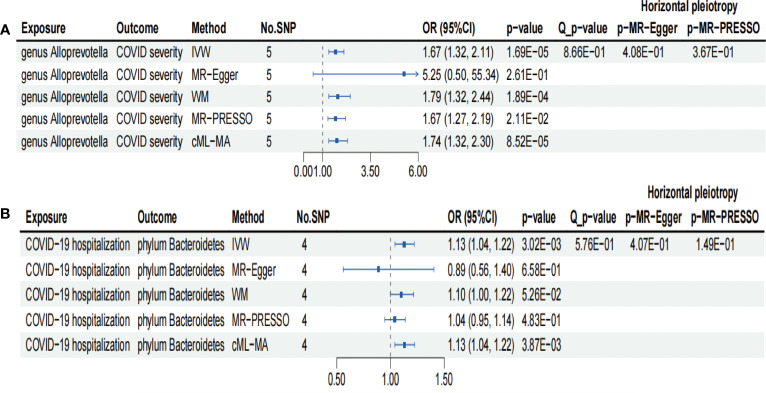
Forests plot of sensitivity analyses for two associations. **(A)** Genus *Alloprevotella* with COVID-19 severity. **(B)** COVID-19 hospitalization with phylum *Bacteroidetes*. IVs, instrumental variables; WM, weighted median; MR-PRESSO, MR pleiotropy residual sum and outlier; cML-MA, constrained maximum likelihood and model averaging-based. E-01 represents 10^−1^.

As shown in **Figure 2A**, IVW analysis revealed that genus *Ruminococcus gnavus* group (OR = 0.77; 95% CI, 0.62–0.95; *p* = 1.44×10^−2^), genus *Oxalobacter* (OR = 0.84; 95% CI, 0.71–1.00; *p* = 4.96×10^−2^), and genus *Ruminiclostridium6* (OR = 0.78; 95% CI, 0.62–0.98; *p* = 3.55×10^−2^) showed suggestive associations with a reduced risk for COVID-19 severity. On the other hand, IVW analysis revealed a causal link between the genus *Alloprevotella* (OR = 1.67; 95% CI, 1.32–2.11; *p* = 1.69×10^−5^) and heightened COVID-19 severity risk after FDR correction (*p*-FDR = 2.01×10^−3^). In addition, the IVW, MR-Egger, WM, MR-PRESSO, and cML-MA methods yielded the similar direction for the causal effect of genus *Alloprevotella* on COVID-19 severity (**Figure 3A**).

According to the results of the IVW analysis presented in **Figure 2B**, genus *Alistipes* (OR = 0.78; 95% CI, 0.63–0.96; *p* = 2.02×10^−2^), genus *Parasutterella* (OR = 0.84; 95% CI, 0.72–0.98; *p* = 3.09×10^−2^), genus *Ruminiclostridium6* (OR = 0.80; 95% CI, 0.69–0.94; *p* = 5.69×10^−3^), and genus *Ruminococcaceae UCG014* (OR = 0.79; 95% CI, 0.65–0.97; *p* = 2.37×10^−2^) showed suggestive associations with a reduced risk for COVID-19 hospitalization. Conversely, family *Family XIII* (OR = 1.30; 95% CI, 1.03–1.64; *p* = 2.69×10^−2^), family *Victivallaceae* (OR = 1.11; 95% CI, 1.00–1.24; *p* = 4.55×10^−2^), genus *Alloprevotella* (OR = 1.25; 95% CI, 1.07–1.45; *p* = 3.71×10^−3^), and genus *Prevotella9* (OR = 1.21; 95% CI, 1.04–1.41; *p* = 1.47×10^−2^) exhibited suggestive associations with an increased risk for COVID-19 hospitalization. However, the aforementioned associations ceased to be statistically significant once they underwent FDR correction (*p*-FDR > 0.05).

We found that phylum *Lentisphaerae* (OR = 0.93; 95% CI, 0.87–0.99; *p* = 3.23×10^−2^), family *Alcaligenaceae* (OR = 0.87; 95% CI, 0.78–0.96; *p* = 8.55×10^−3^), family *Lachnospiraceae* (OR = 0.91; 95% CI, 0.84–1.00; *p* = 4.46×10^−2^), genus *Dialister* (OR = 0.91; 95% CI, 0.82–1.00; *p* = 4.33×10^−2^), genus *Parasutterellaon* (OR = 0.89; 95% CI, 0.83–0.97; *p* = 4.00×10^−3^), genus *Ruminococcaceae UCG003* (OR = 0.90; 95% CI, 0.82–0.99; *p* = 3.58×10^−2^), and genus *Ruminococcaceae UCG014* (OR = 0.88; 95% CI, 0.80–0.97; *p* = 8.97×10^−3^) showed suggestive associations with a reduced risk for COVID-19 infection using the IVW method (**Figure 2C**). On the other hand, class *Negativicutes* (OR = 1.13, 95% CI, 1.02–1.26; *p* = 2.45×10^−2^), order *Selenomonadales* (OR = 1.13; 95% CI, 1.02–1.26; *p* = 2.45×10^−2^), and genus *Phascolarctobacterium* (OR = 1.13; 95% CI, 1.02–1.25; *p* = 1.92×10^−2^) exhibited suggestive associations with a higher risk for COVID-19 infection (**Figure 2C**). After FDR correction, the aforementioned associations lost their statistical significance (*p*-FDR > 0.05).

### 3.2 Causal effects of COVID-19 phenotypes on gut microbiome

In the reverse MR (COVID-19 as the exposure), we identified 1233, 827 and 1033 SNPs associated with COVID-19 severity, hospitalization, and infection respectively. All SNPs of gut microbiome included in the analysis had an F-statistic greater than 10 (**Table S4**), suggesting the absence of weak bias of IVs. Additionally, the adjusted IVW results, which considered heterogeneity, are presented in **Table S5**.

**Figure 4:**
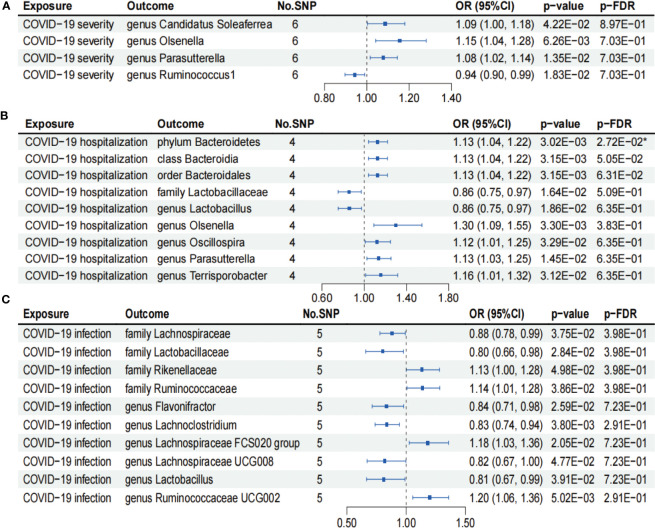
Forests plot of causal effects of COVID-19 phenotypes on gut microbiome (*p*-IVW > 0.05). **(A)** COVID-19 severity. **(B)** COVID-19 hospitalization. **(C)** COVID-19 infection. FDR, false discovery rate. * *p*-FDR < 0.05. E-01 represents 10^−1^.

As shown in **Figure 4A**, IVW analysis suggested that COVID-19 severity showed a suggestive association with a decreased abundance of the genus *Ruminococcus1* (OR = 0.94; 95% CI, 0.90–0.99; *p* = 1.83×10^−2^). In contrast, COVID-19 severity exhibited suggestive associations with an increased abundance of the genus *Candidatus Soleaferrea* (OR = 1.09; 95% CI, 1.00–1.18; *p* = 4.22×10^−2^), genus *Olsenella* (OR = 1.15; 95% CI, 1.04–1.28; *p* = 6.26×10^−3^), and genus *Parasutterella* (OR = 1.08; 95% CI, 1.02–1.14; *p* = 1.35×10^−2^). Nevertheless, the aforementioned associations between COVID-19 severity and gut microbiome lost their statistical significance after undergoing FDR correction (*p*-FDR > 0.05).

As presented in **Figure 4B**, IVW analysis suggested that COVID-19 hospitalization showed suggestive associations with a decreased abundance of the family *Lactobacillaceae* (OR = 0.86; 95% CI, 0.75–0.97; *p* = 1.64×10^−2^) and genus *Lactobacillus* (OR = 0.86; 95% CI, 0.75–0.97; *p* = 1.86×10^−2^). On the other hand, COVID-19 hospitalization exhibited suggestive associations with a higher abundance of the class *Bacteroidia* (OR = 1.13; 95% CI, 1.04–1.22; *p* = 3.15×10^−3^), order *Bacteroidales* (OR = 1.13; 95% CI, 1.04–1.22; *p* = 3.15×10^−3^), genus *Oscillospira* (OR = 1.12; 95% CI, 1.01–1.25; *p* = 3.29×10^−2^), genus *Parasutterella* (OR = 1.13; 95% CI, 1.03–1.25; *p* = 1.45×10^−2^), and genus *Terrisporobacter* (OR = 1.16; 95% CI, 1.01–1.32; *p* = 3.12×10^−2^). Notably, COVID-19 hospitalization was causally associated with an increased abundance of phylum *Bacteroidetes* (OR = 1.13; 95% CI, 1.04–1.22; *p* = 3.02×10^−3^), even after FDR correction (*p*-FDR = 2.72×10^−2^).

We found that COVID-19 infection showed suggestive associations with a decreased abundance of the family *Lachnospiraceae* (OR = 0.88; 95% CI, 0.78–0.99; *p* = 3.75×10^−2^), family *Lactobacillaceae* (OR = 0.80; 95% CI, 0.66–0.98; *p* = 2.84×10^−2^), genus *Flavonifractor* (OR = 0.84; 95% CI, 0.71–0.98; *p* = 2.59×10^−2^), genus *Lachnoclostridium* (OR = 0.83; 95% CI, 0.74–0.94; *p* = 3.80×10^−3^), genus *Lachnospiraceae UCG008* (OR = 0.82; 95% CI, 0.67–1.00; *p* = 4.77×10^−2^), and genus *Lactobacillus* (OR = 0.81; 95% CI, 0.67–0.99; *p* = 3.91×10^−2^) using the IVW analysis (**Figure 4C**). In contrast, COVID-19 infection exhibited suggestive associations with an increased abundance of the family *Rikenellaceae* (OR = 1.13; 95% CI, 1.00–1.28; *p* = 4.98×10^−2^), family *Ruminococcaceae* (OR = 1.14; 95% CI, 1.01–1.28; *p* = 3.86×10^−2^), genus *Lachnospiraceae FCS020 group* (OR = 1.18; 95% CI, 1.03–1.36; *p* = 2.05×10^−2^), and genus *Ruminococcaceae UCG002* (OR = 1.20; 95% CI, 1.06–1.36; *p* = 5.02×10^−3^) (**Figure 4C**). However, the aforementioned associations between COVID-19 infection and gut microbiome failed to pass the FDR correction test (*p*-FDR > 0.05).

### 3.3 Sensitivity analyses

**Figure 5:**
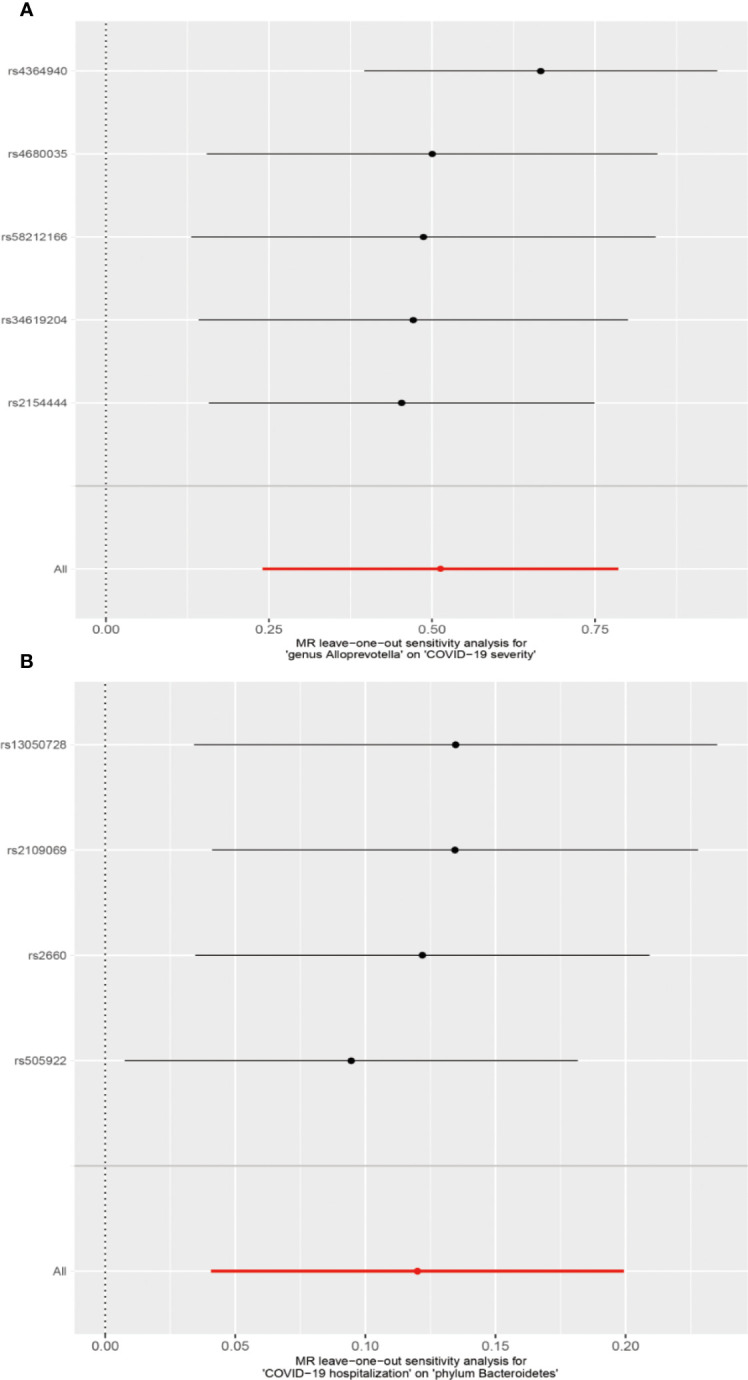
MR leave-one-out sensitivity analysis for two associations. **(A)** Genus *Alloprevotella* with COVID-19 severity. **(B)** COVID-19 hospitalization with phylum *Bacteroidetes*.

We conducted several sensitivity analyses to evaluate the robustness of the MR estimates of the two associations which passed the FDR correction test (genus *Alloprevotella* with COVID-19 severity; COVID-19 hospitalization with phylum *Bacteroidetes*; **Figure 3**). Results from the MR-Egger test and MR-PRESSO global test indicated the absence of horizontal pleiotropy (*p*-MR-Egger > 0.05 and *p*-MR-PRESSO > 0.05) in the two associations. The MR-PRESSO analysis revealed that there were no outlier SNPs in the MR results. However, MR-Egger analysis suggested a different direction for the causal estimate of COVID-19 hospitalization on the phylum *Bacteroidetes* compared to IVW, WM, MR-PRESSO, and cML-MA analyses (**Figure 3B**). Therefore, the result of causal estimate of COVID-19 hospitalization on phylum *Bacteroidetes* requires careful consideration. Additionally, the “leave-one-out analysis” indicated that excluding any individual SNP did not significantly alter the overall results of the two associations (**Figure 5**).

## 4 Discussion

The association between the gut microbiome and COVID-19 has been of great interest. In the study, we conducted the bi-directional MR analysis to assess the causal effects and casual directions between gut microbiome and COVID-19 phenotypes. We identified 45 links between gut microbiome and COVID-19 phenotypes, of which 43 were suggestive links and two were strong links. Following the FDR correction, we found that the genus *Alloprevotella* was causally related with a higher risk of COVID-19 severity, while COVID-19 hospitalization was causally linked to an increase in the abundance of the phylum *Bacteroidetes*. As far as we know, this is the first bidirectional MR study to comprehensively investigate the causal association between gut microbiome and COVID-19.

It has suggested that the gut microbiome could modulate the host’s immune system, potentially affecting the disease process of COVID-19 (3, 10, 11). In this study, we found that several bacterial taxa, including the phylum *Lentisphaerae* (infection), family *Alcaligenaceae* (infection), family *Lachnospiraceae* (infection), genus *Ruminococcus gnavus* group (severity), genus *Oxalobacter* (severity), genus *Ruminiclostridium6* (severity, hospitalization), genus *Alistipes* (hospitalization), genus *Parasutterella* (hospitalization, infection), genus *Ruminococcaceae UCG003* (infection), genus *Ruminococcaceae UCG014* (hospitalization, infection), and genus *Dialister* (infection), had suggestive protective effects against COVID-19 phenotypes. Short-chain fatty acids (SCFAs), which belonged to immunomodulatory metabolites, played a vital role in alleviating pulmonary diseases (56). SCFAs might regulate lung immunity through the following mechanisms (10). First, SCFAs directly migrated to lung tissues through the circulation and exerted regulatory effects on pulmonary immunity (10). Second, SCFAs stimulated the differentiation and activation of B cells, leading to the production of immunoglobulin A. In the lung, immunoglobulin A facilitated the clearance of viruses (57). Third, SCFAs enhanced the differentiation and activation of Treg cells, which produced IL-10 and TGF-β, thereby reducing lung inflammation and injury (58, 59). Several studies revealed that the above bacteria, including the family *Lachnospiraceae* (14), genus *Ruminococcus gnavus group* (60), genus *Alistipes* (14), and genus *Parasutterella* (61), had the capability to produce short-chain fatty acids and exerted an anti-inflammatory effect, thereby potentially alleviating COVID-19 symptoms (3, 14). Interestingly, clinical trials have demonstrated that supplementing with the family *Lachnospiraceae* could be an effective way to enhance recovery from COVID-19 and alleviate associated symptoms (26, 62, 63). On the other hand, the family *XIII* (hospitalization), family *Victivallaceae* (hospitalization), class *Negativicutes* (infection), order *Selenomonadales* (infection), genus *Alloprevotella* (hospitalization), genus *Prevotella9* (hospitalization), and genus *Phascolarctobacterium* (infection) were found to have suggestive contributory effects on COVID-19 phenotypes. Specifically, the IVW analysis suggested that the genus *Alloprevotella* was casually associated with a higher risk of COVID-19 severity after FDR correction. In addition, it was found that the genus *Alloprevotella* was enriched in COVID-19 hospitalized patients at the nasopharynx (64, 65). Previous studies have suggested that genus *Prevotella9* (66), genus *Alloprevotella* (64, 67), and genus *Phascolarctobacterium* (68) exhibited increased inflammatory properties and were thought to be clinically important pathobionts involved in promoting chronic inflammation. This might explain why these bacteria pose a risk for COVID-19. Taken together, these findings highlight the significance of gut microbiome as a modifiable factor in enhancing the outlook of COVID-19.

Previous research has revealed that COVID-19 patients often suffer from various gastrointestinal reactions (8, 9). In this study, IVW analysis revealed that COVID-19 phenotypes could potentially reduce the abundance of the family *Lactobacillaceae* (hospitalization, infection), family *Lachnospiraceae* (infection), genus *Ruminococcus1* (severity), genus *Lactobacillus* (hospitalization, infection), genus *Flavonifractor* (infection), genus *Lachnoclostridium* (infection), and genus *Lachnospiraceae UCG008* (infection). In addition, COVID-19 phenotypes potentially increased the abundance of the class *Bacteroidia* (hospitalization), order *Bacteroidales* (hospitalization), family *Rikenellaceae* (infection), family *Ruminococcaceae* (infection), genus *Candidatus Soleaferrea* (severity), genus *Olsenella* (severity, hospitalization), genus *Parasutterella* (severity, hospitalization), genus *Oscillospira* (hospitalization), genus *Terrisporobacter* (hospitalization), genus *Lachnospiraceae FCS020 group* (infection), and genus *Ruminococcaceae UCG002* (infection). Notably, COVID-19 hospitalization was found to be casually associated with an increased abundance of the phylum *Bacteroidetes* after FDR correction by the IVW analysis, although MR-Egger suggested a different causal direction. Recent studies have also observed a decrease in the abundance of the family *Lachnospiraceae* (69) and an increase in the abundance of the phylum *Bacteroidetes* (15, 70–72), family *Ruminococcaceae* (69, 73), and genus *Oscillospira* (16) subsequent to SARS-CoV-2 infection. We therefore deem that COVID-19 may exacerbate disease symptoms by disrupting the gut microbiota homeostasis. Generally speaking, these findings support the notion that COVID‐19 has impacts on gut microbiome dysbiosis through the “gut-lung axis”.

In the study, we observed a notable correlation between the gut microbiome residing in the gastrointestinal tract and the clinical outcomes of SARS-CoV-2 infection. Additionally, we discovered that SARS-CoV-2 infection had the potential to induce modifications in the gut microbiome. These findings provided support for the bidirectional interaction between the gut and the lung known as the “gut-lung axis” (74). The gut microbiome reportedly played a crucial role in modulating immune responses in the lung (3, 10). An underlying mechanism by which the gut microbiome contributed to influencing the outcomes of COVID-19 is via the activities of its metabolites (e.g., SCFAs) (11, 29). The potential exists for the gut microbiome, along with its metabolites, to impact the gene expression of type I interferon (IFN-I) receptors in respiratory epithelial cells. This, in turn, could restrict the proliferation of influenza viruses by stimulating the production of IFN-α and IFN-β (75, 76). Furthermore, the metabolites originating from the gut microbiome had the capacity to stimulate the migration of dendritic cells from the lung to the draining lymph node and promote T-cell priming through the activation of inflammasomes (77). On the other hand, the gut microbiome stimulated the release of inflammatory factors that disseminated throughout the body, exerting their effects on various mucosal tissues and exacerbating the cytokine storm, thereby exacerbating the severity of the condition (23). In this study, we observed a causal association between the genus *Alloprevotella* and an elevated risk of severe COVID-19. A study has indicated a positive correlation between the abundance of genus *Alloprevotella* and the level of C-reactive protein, a well-known marker of inflammation (78). We hypothesized that the genus *Alloprevotella* could promote inflammation and, thus, exacerbate the disease. However, research focused on the direct influence of the gut microbiome in COVID-19 remains limited.

SARS-CoV-2 has been suggested to potentially spread from the lung through transportation via immune cells within the circulatory and lymphatic systems (3, 23). Direct infection of gut epithelial cells by SARS-CoV-2 compromised the integrity of the gut barrier and facilitated microbial translocation. This cascade set off a cytokine storm, exacerbating dysregulation in the gut microbiome, metabolites, electrolytes, and gut barrier functions (3, 79). The invasion of SARS-CoV-2 could trigger the activation of pattern-recognition receptors, which were recognized by innate immune cells. This activation led to the release of diverse pro-inflammatory cytokines (80). These immune responses, once activated, could potentially impair gut permeability, disturb the equilibrium of gut microbiome, and lead to an increase in opportunistic pathogens (e.g., *Bacteroidetes*) and a decrease in commensal symbionts (e.g., *Lactobacillus*). On the other hand, ACE2 receptors in the gut appeared to be a critical factor in mediating the interaction between SARS-CoV-2 and the gut microbiome. SARS-CoV-2 bound to the ACE2 receptor, leading to a decrease in ACE2 receptor concentration and consequently in the diversity of the gut microbiome (3, 74, 81). COVID-19 has been shown to impact the gut microbiome in this study, specifically phylum *Bacteroidetes*, potentially through these mechanisms.

There are several interventions based on the gut microbiome that show potential for addressing COVID-19. First, the gut microbiome can serve as biomarkers for predicting the prognosis of COVID-19. Previous studies have suggested an association between the gut microbiome and the prognosis of COVID-19 (17–19). The microbiota associated with COVID-19, identified in this study, can also be considered as markers for predicting disease progression in COVID-19 (82). Second, a meta-analysis of 1198 patients indicated that probiotics could alleviate symptoms and immune responses and reduce the duration of symptoms in patients with COVID-19 (83). These findings demonstrate the ability of probiotics to effectively reshape gut microbiome homeostasis and reduce inflammatory responses, ultimately acting as adjuvants against SARS-CoV-2. Third, fecal transplants might represent a safe intervention to alleviate gastrointestinal symptoms and modulate immune responses (84, 85). Fourth, meta-analysis indicated that bacterial co-infections were infrequent and that widespread antibiotic usage did not improve the clinical outcome of COVID-19 (86). Therefore, it is crucial to implement antimicrobial stewardship to prevent antibiotic-induced dysbiosis of the gut microbiota and mitigate the risks of disease severity and antimicrobial resistance (87, 88).

This study exhibited several strengths. First, this study utilized the largest publicly available GWAS data of gut microbiome and COVID-19 from over 1.8 million individuals with different ethnicities, providing reliable evidence to elucidate the association between gut microbiome and COVID-19. Second, previous epidemiological studies might be prone to biases due to confounders or reverse causation, but the MR design could effectively minimize these biases. Third, stringent quality control procedures and multiple sensitivity analysis approaches were employed in this study to assess the robustness of the MR estimates (30). Fourth, the potential links identified in this study could assist in further investigations into the mechanisms underlying the links between gut microbiome and COVID-19 phenotypes.

The study’s limitations, nevertheless, must be acknowledged. Due to a lack of sufficient SNPs (less than three) after linkage disequilibrium, we relaxed the *p*-value threshold (*p* lower than 1.0×10^–5^) of SNPs of gut microbiome (gut microbiome as the exposure) in accordance with previous studies (41–47), which might result in weak instrumental variables. To address this issue, we calculated the F-statistics to measure the power of each SNP. All SNPs used in the study having an F-statistic greater than 10 indicated the absence of weak instrument bias. Additionally, bacterial taxa at the species level were not available. Further research is required to elucidate the causal links between the species of gut microbiome and COVID-19 phenotypes.

By conducting the bi-directional MR analysis using the publicly available GWAS summary data, we comprehensively explored the causal link between gut microbiome and COVID-19. This study revealed the interaction between gut microbiome and COVID-19 through the “gut-lung axis”. These findings support the notion that the gut microbiome can serve as an intervention target and may offer new insights into preventing, diagnosing and treating COVID-19.

## Data availability statement

The original contributions presented in the study are included in the article/**Supplementary Materials**. Further inquiries can be directed to the corresponding authors.

## Ethics statement

Ethical review and approval was not required for the study on human participants in accordance with the local legislation and institutional requirements. The patients/participants provided their written informed consent to participate in this study.

## Author contributions

ZL, GZ, LZ, and LS conceived and designed the study. ZL and GZ wrote the original manuscript, analyzed the data, and drew the figures. LZ and LS supervised the study with funding support. LZ and LS took responsibility for the contents of the article. All authors critically reviewed and revised the manuscript. All authors contributed to the article and approved the submitted version.
